# Meshless treatment of open inguinal hernia repair: a prospective study

**DOI:** 10.1590/S1679-45082013000200009

**Published:** 2013

**Authors:** Paulo Kassab, Ettore Ferrari Franciulli, Carolina Kassab Wroclawski, Elias Jirjoss Ilias, Osvaldo Antônio Prado Castro, Carlos Alberto Malheiros

**Affiliations:** 1 Faculdade de Ciências Médicas da Santa Casa de São Paulo, São Paulo, SP, Brazil; 2 Hospital Wladimir Arruda, São Paulo, SP, Brazil; Postgraduate Program, Faculdade de Ciências Médicas da Santa Casa de São Paulo, São Paulo, SP, Brazil; Hospital Wladimir Arruda, São Paulo, SP, Brazil; Postgraduate Program, Faculdade de Ciências Médicas da Santa Casa de São Paulo, São Paulo, SP, Brazil; 3 Faculdade de Medicina do ABC, Santo André, SP, Brazil

**Keywords:** Hernia, inguinal/surgery, Digestive system surgical procedures/methods, Suture techniques, Recurrence

## Abstract

**Objective:**

To evaluate two types of meshless open inguinal repair and to evaluate the recurrence rate.

**Methods:**

We operated on sequentially 98 men and 15 women with 144 unilateral or bilateral inguinal hernias between December 1988 and April 2007. The surgeries were performed by two experienced surgeons and divided into two groups: Bassini or McVay reconstructive surgery techniques. Bassini type reinforcements were employed for Nyhus II and IIIB with minor destruction of the posterior wall. Patients with Nyhus type IIIA, type IIIB with major destruction of the fascia transversalis, and type IIIC were subjected to the McVay technique.

**Results:**

Seventy-five hernias were corrected using the McVay technique. Only two recurrences (2.67%) were observed in this group. For group Bassini, two recurrences for 69 hernias (2.89%) were observed (p=0.658). Mean age for the recurrent group was 56 years. No differences were observed between the ages of males and females (52 years).

**Conclusions:**

Non-mesh repair in inguinal hernia can be safely used if performed by experienced surgeons.

## RESUMO

**Objetivo:**

Avaliar dois tipos de correção de hérnias inguinais sem o uso de tela e avaliar a taxa de recidiva.

**Métodos:**

Foram operados sequencialmente 98 homens e 15 mulheres portadores de 144 hérnias inguinais unilaterais ou bilaterais entre dezembro de 1988 e abril de 2007. As cirurgias foram realizadas por dois cirurgiões experientes e divididas em dois grupos: reconstruções pela técnica de Bassini ou McVay. Os reforços tipo Bassini foram utilizados para hérnias do tipo Nyhus II e IIIB, em que havia menor destruição da parede posterior do canal inguinal. Os pacientes com hérnias do tipo Nyhus IIIA e Nyhus IIIB, em que havia maior destruição da fáscia transversalis, e Nyhus IIIC foram submetidos à técnica de McVay.

**Resultados:**

Setenta e cinco hérnias foram corrigidas utilizando-se a técnica de McVay. Somente duas recidivas (2,67%) foram observadas nesse grupo. No grupo Bassini, foram observadas duas recidivas (2,89%) entre 69 hérnias operadas (p=0,658). A média de idade para o grupo de pacientes com recidiva foi de 56 anos. Não foram observadas diferenças de idades entre homens e mulheres (52 anos).

**Conclusão:**

Os reparos sem telas nas hérnias inguinais podem ser seguramente utilizados, se realizados por cirurgiões experientes.

## INTRODUCTION

There is no consensus regarding the “best” surgical treatment of hernia, despite the general belief on the opposite. Many reasons, including economic factors, led to the publication that the use of tensionless prostheses had resolved inguinal hernia surgery recurrence. Notwithstanding – and based on medical publications^(1–4)^ and on our own professional practice – we have witnessed that recurrence persists with or without the use of meshes, together with new prosthesis-related problems, which include chronic pain and oligospermia. For developing nations, these issues represent an additional surgical cost.

Systematic research^(5,6)^, with continuing patient follow-up, has evidenced very similar recurrence, with and without the use of meshes, when surgery is undertaken by inexperienced professionals.

Published researches^(6)^ often refers to teaching hospitals where, in general (at least in Brazil), this surgery is performed by residents. This finding is an important source of bias, since systematic scholarly research has revealed very similar levels of recurrence, with and without the use of meshes, when the surgery is performed by experienced professionals.

With the intent of addressing this bias and minimizing the effect of possible technical failures, we designed a prospective sequential study with patients from private surgical center.

## OBJECTIVE

To evaluate McVay and Bassini techniques in open hernia repair concerning the recurrence rate and to evaluate the recurrence rate.

## METHODS

A total of 98 men and 15 women, with 144 unilateral or bilateral inguinal hernias, were prospectively and sequentially evaluated between December 1988 and April 2007. Patients were selected from private practice office and operated on sequentially at different private hospitals.

Patients' age ranged from 19 to 87 years. The mean age of the 113 individuals was 54.52 years.

Individuals were divided into two groups and treated with either Bassini or McVay reconstructive surgical techniques. The hernias were classified according to the Nyhus classification system: (a) type I – indirect hernias with a deep inguinal ring of normal diameter; (b) type II – indirect hernias with enlargement of the deep inguinal ring; (c) type III – presence of defects in the posterior wall of the inguinal canal, subdivided into A, B and C; (d) type IIIA – direct inguinal hernia; (e) type IIIB – indirect inguinal hernia with a weakening of the posterior wall; (f) type IIIC – femoral hernia; (g) type IV – recurrent hernias.

All surgical procedures were performed by the same clinical team at private hospitals. Cephalexin was administered, on a prophylactic basis, during the surgical procedure. Post-surgical follow-up was undertaken by the responsible surgeon during a period ranging from 10 to 208 months of follow-up. The patients were evaluated individually by the responsible surgeon. Only physical examination was performed, and no complementary image evaluation was done.

The following parameters were analyzed: age, gender, type of hernia, location and recurrence.

### Inclusion and exclusion criteria

Patients afflicted with primary inguinal hernia who were older than 18 years of age were included in the study. Patients with recurrent hernias or under 18 years were excluded.

The Bassini type surgery was employed for Nyhus II and IIIB with minor destruction of the posterior wall^(7)^. The most important suture criterion was the condition of the transversalis fascia. Patients with Nyhus type IIIA, type IIIB with major destruction of the transversalis fascia, and type IIIC were subjected to the McVay technique. Reinforcements were always made with separate polyester and cotton (Polycot^®^) suture. All sutures were always completed without tensioning and, if necessary, a relaxing incision was made laterally to the rectus muscle sheath.

### Statistical analysis

The χ^2^ test and Fisher's exact test were used to compare group proportions (with and without recurrence, men and women) and to evaluate the association between independent observations. Null hypotheses were rejected at the 0.05 or 5% level of significance.

This study was approved by the Research Ethics Committee of the institution *Irmandade da Santa Casa de Misericórdia de São Paulo* under number # 361/09.

## RESULTS

Seventy-five hernias were repaired using the McVay technique, and only two recurrences (2.67%) were observed for this group. For the group repaired using the Bassini technique, two recurrences for 69 hernias (2.89%) were observed (p=0.658). Mean age for the recurrent group was 56 years. No differences were observed between the age of men and women (52 years) (Table 1).

**Table 1:** Incidence of recurrence and gender

Gender	Recurrence				Total	
	Yes		No		n	%
	n	%	n	%		
Male	4	100	125	89	129	3.10
Female	0	0	15	11	15	0
Total	4	100	140	100	144	2.78

Fisher's exact test; p=0.684 (ns)

We did not observe differences in recurrence rate between men and women; nevertheless, the recurrence rate was higher when the patient had associated diseases (Tables 2 and 3).

**Table 2:** Relationship of Nyhus type and recurrence

Type of hernia	Recurrence				Total	
	Yes		No		n	%
	n	%	n	%		
II	0	0	39	28	39	0
IIIA	2	50	66	47	68	2.94
IIIB	2	50	32	23	34	5.88
IIIC	0	0	3	2	3	0
Total	4	100	140	100	144	2.78

**Table 3:** Surgical procedure and Nyhus type

Nyhus	Surgical technique		Total	
	B	MV	n	%
II	39	0	39	27.1
IIIA	2	67	69	48
IIIB	28	5	33	23
IIIC	0	3	3	20.1
Total	69	75	144	100

χ^2^ test = 112.3*; p<0.01 B Technique > Nyhus II e IIIB, MV Technique > Nyhus IIIA e IIIC.

B: Bassini technique; MV: McVay technique.

We did not observe differences related to type of surgery.

## DISCUSSION

Although many advances have taken place for the repair of inguinal hernia, there is still a discussion regarding the effectiveness of the ideal technique to be used during surgical procedures. It is known that nowadays the mesh repair is widely and safely used with better results despite concerns about chronic pain^(8)^. A randomized trial concluded that laparoscopic techniques also showed better results when compared with Lichtenstein's repair^(9)^. On the other hand, another randomized trial showed no benefits of laparoscopic and mesh techniques when compared with meshless surgery^(10)^.

Several studies have revealed that recurrence increases when prostheses are not employed. Mittelstaedt et al.^(11)^, in a randomized prospective study, used the classical Bassini, McVay and Shouldice techniques to correct 136 hernias in 119 patients. Forty-four patients were treated with the Bassini technique; fifty with the McVay and 42 patients with the Shouldice technique. After a four-year follow-up, recurrence was diagnosed for eight patients (35.7%) operated with the Bassini technique, for two patients (8.5%) with the McVay technique, and three patients (23.7%) with the Shouldice technique.

Despite of the 9.55% recurrence level, we believe that the use of random assignment and the application of inadequate techniques, such as the Bassini for type IIIA Nyhus hernia, were also a contributing factor. No information was provided regarding the number and experience members participating in the surgical team. Finally, we consider that a four-year follow-up is unduly short and, for three recurrences (two using the Bassini and one with McVay technique), there were local complications (wound infection), which could probably be avoided through the use of appropriate anti-bacterial prophylaxis.

In yet another comparison of the Bassini technique with prostheses, published by Witkolowski et al.^(12)^, unfavourable results were observed for 20.5% and 9.4% respectivelly of the subjects. Most surgery was undertaken by different surgeons who were primarily residents. For 16 Bassini technique recurrences, three developed coughing due to the pneumonia acquired during the post-operative period. Three surgical procedures were undertaken for recurrent hernia, and five emergency procedures were carried out for incarcerated hernia.

It is surprising that Bassini, in his classical publication in 1894, reported that out of 206 surgical procedures undertaken for inguinal hernia repair which included emergency procedures to resolve strangulation, a low recurrence rate was seen. His patients were monitored during 5 years. Results were surprising, since there was no surgical demise and only eight recurrences were reported^(7)^. Why then did Bassini achieve these results, which are far better than contemporary authors, although modern technological resources and materials were unavailable at that time?

We believe that a number of factors contributed to Bassini's results. First of all is the widespread belief that inguinal hernia surgery is a simple procedure that should be performed by younger less-experienced surgeons. The heterogeneity of surgical teams is yet another factor evidenced in teaching hospitals, where a large numbers of surgeons and residents are found. In our opinion, a lack of anatomical expertise and inguinal hernia physiopathology is notable in the young learners. Inadequate orientation regarding the required preventive post-operative measures after discharge, the lack of good common sense when choosing the best surgical technique, and because of the belief that to master one or two techniques seem to be enough for all inguinal hernia are also contributing factors^(5,6)^.

Our results reveal that, in general, type II and even type IIIB Nyhus, without relevant destruction of the transversalis fascia can be corrected using the Bassini technique. We propose that types IIIA or type IIIB with large destruction of the transversalis fascia should be treated using the McVay technique. This study revealed that age and gender do not affect recurrence. This result requires further study, particularly for the reduced number of female patients who participated in our study.

The discussion regarding tensionless suture is quite old, and it is an accepted fact that tensioned reinforcement is condemned to recurrence^(13)^. Notwithstanding, an experienced surgeon is normally capable of employing tensionless suturing, with and without the use of meshes. To accomplish this, the surgeon can employ relaxing incisions next to the lateral of the rectus muscle. We are concerned about the ability of younger surgeons to perform meshless repairs because it is rare, even in teaching hospitals, to see this type of surgery. It seems that there is abuse in indications for the use of prosthesis in hernia surgery^(14)^.

Mesh surgery is normally more expensive, although they are now more accessible. In Brazil, for example, the public service pay more for the prosthesis than for the surgeon^(14,15)^. Another disadvantage indeed is the existence of infection and the chronic pain, even though many surgeons do not report such events. In general, the use of prosthesis in women, particularly in the young ones, is not necessary because their transversalis fascia is often preserved^(14)^.

In our opinion, the Bassini and McVay are still suitable and, when properly prescribed and completed, they constitute the best choice of simple and inexpensive surgery, with low complication and recurrence rates.

Consequently, the socio-economic cost impact is low. This could be important particularly in rural areas and in poor countries. Other ecological benefits, as the reduction of the use of plastic materials, can be obtained.

## CONCLUSIONS

Bassini e McVay techniques can still be used to repair inguinal hernia due to low complication and recurrence rates. The Bassini technique should be employed for type II and IIIB hernias with reduced destruction of the transversalis fascia, and the McVay technique should be preferably employed for type IIIA, IIIC hernias and in type IIIB in which major destruction of the transversalis fascia has occurred.
